# USP30-AS1 Dictates Breast Cancer Cell Fate and Chemoresistance via a miR-3646/FZD7/Wnt/β-Catenin Circuit

**DOI:** 10.3390/cimb47120974

**Published:** 2025-11-24

**Authors:** Qian He, Shiyue Yang, Qilei Xin, Yapei Jiang, Li Jiang, Naihan Xu

**Affiliations:** 1 School of Food and Drug, Shenzhen Polytechnic University, Shenzhen 518055, China; 2 State Key Laboratory of Chemical Oncogenomics, Institute of Biopharmaceutical and Health Engineering, Shenzhen International Graduate School, Tsinghua University, Shenzhen 518055, China

**Keywords:** long non-coding RNA, breast cancer, stem cells, chemoresistance, MicroRNA, WNT pathway

## Abstract

Cancer stem cells (CSCs) play a pivotal role in promoting tumorigenesis, drug resistance, invasion, and metastasis. Recent studies indicate that long non-coding RNAs (LncRNAs) directly or indirectly regulate CSCs, influencing tumor progression. This study investigated the role of LncRNA USP30-AS1 in maintaining stemness and chemoresistance in breast cancer. USP30-AS1 was significantly upregulated in BCSC-enriched mammospheres derived from MDA-MB-231 and MCF-7 cell lines, where it correlated with elevated stemness markers (CD44, ALDH1A1, OCT4) and an increased proportion of ALDH+ cells. Functional experiments demonstrated that knockdown of USP30-AS1 reduced spheroid formation, stemness marker expression, chemoresistance, migration, and invasion, while its overexpression promoted these phenotypes. Mechanistically, USP30-AS1 acts as a competing endogenous RNA (ceRNA) by sponging miR-3646, which leads to the derepression of Frizzled-7 (FZD7) and subsequent activation of the Wnt/β-catenin signaling pathway. These findings identify USP30-AS1 as a critical promoter of stemness, chemoresistance, and metastasis in BCSCs via the miR-3646/FZD7/Wnt axis, suggesting it is a potential therapeutic target for breast cancer intervention.

## 1. Introduction

Breast cancer remains the most frequently diagnosed malignancy and leading cause of cancer-related mortality among women worldwide. While advances in targeted therapies have improved clinical outcomes, tumor recurrence and metastasis driven by breast cancer stem cells (BCSCs) continue to pose major therapeutic challenges [1]. BCSCs constitute a small yet powerful subpopulation within tumors, characterized by self-renewal capacity, limited proliferation potential, and intrinsic chemoresistance. They are responsible for 40–70% of recurrence events [2,3]. These cells evade conventional treatment through various mechanisms, including quiescence, enhanced DNA damage repair, ABC transporter-mediated drug efflux, and adaptation to hypoxic microenvironments [4,5,6,7]. BCSCs are commonly identified by elevated expression of stemness markers such as CD44+/CD24−, ALDH1, and SOX2, which correlate strongly with therapeutic resistance and poor prognosis [8,9,10]. Overcoming BCSC-mediated resistance is thus essential to improve long-term survival in breast cancer patients.

Long non-coding RNAs (lncRNAs) are a class of non-coding RNAs over 200 nucleotides long. They play pivotal roles in regulating genome architecture and gene expression [11]. By binding with DNA, RNA, or proteins, lncRNAs modulate gene expression at multifaceted levels, including chromatin remodeling, splicing, transcriptional and post-transcriptional regulation [12,13]. LncRNAs are distributed in both the nucleus and cytoplasm. Nuclear-localized lncRNAs often influence transcriptional processes by interacting with epigenetic modifiers, transcriptional factors, and spliceosomal components. In the cytoplasm, lncRNAs modulate mRNA stability and translation by serving as competing endogenous RNAs [14]. Aberrant expression of lncRNAs has been frequently observed in various of human cancers, where they contribute to cancer development and progression by influencing key cellular processes such as cell proliferation, migration, invasion, apoptosis, epithelial–mesenchymal transition (EMT), stemness, and drug resistance [15,16,17]. In breast cancer, a variety of lncRNAs exhibit significant expression divergence across different molecular subtypes, making them promising predictive biomarkers and attractive therapeutic targets [18,19]. Recent studies have shown that certain lncRNAs are abnormally expressed in BCSCs, where they regulate key signaling pathways involved in tumor initiation and progression. For instance, lnc RNA HOTAIR is overexpressed in BCSCs and promotes cancer stemness by modulating EMT via the HoxD10/miR-7/SETDB1/STAT3 axis and concurrently activating NF-κB signaling [20,21]. Similarly, lncRNA H19, which is upregulated in BCSCs, maintains cancer stemness by sponging miRNR let-7, thereby enhancing the expression of LIN28 and ESR1 and subsequent activation of the Wnt pathway [22]. Additionally, lncRNA XIST enhances the self-renewal capacity of BCSCs by activating pro-inflammatory IL-6/STAT3 signaling [23].

USP30-AS1 (Ubiquitin-Specific Peptidase 30 Antisense RNA 1) is transcribed from the antisense strand of USP30 and has been implicated in the development and progression of multiple cancer types [24,25,26,27,28]. For instance, in cervical cancer, USP30-AS1 facilitates progression by sponging miR-299-3p and upregulating PTP4A1 [29]; in glioblastoma, it promotes growth by suppressing mitophagy [25]; and in acute myeloid leukemia, it enhances cell survival by cic-regulating USP30 and ANKRD13A [26]. Our group has previously reported that USP30-AS1 suppresses inflammatory signaling in colon cancer via the NF-κB/MYBBP1A pathway [30]. More recently, we demonstrated that USP30-AS1 is significantly upregulated in breast cancer, particularly in triple-negative subtype, where it promotes tumor proliferation through both the hnRNP-F/P21 and EZH2/c-Myc/p21 axes [31]. Importantly, USP30-AS1 exhibits both nuclear and cytoplasmic localization, with cytoplasmic accumulation suggesting potential role as a competing endogenous RNA (ceRNA).

BCSCs frequently depend on hyperactivated signaling pathways such as Wnt/β-catenin for self-renewal and survival [32,33,34]. Constitutive activation of this pathway leads to upregulation of multidrug resistance gene 1 (MDR1) and core stemness factors including NANOG and SOX2 [35,36], directly contributing to therapy failure. In this study, we investigate the role of USP30-AS1 in BCSC maintenance and chemoresistance. We show that USP30-AS1 is markedly upregulated in BCSC-enriched populations and is associated with activation of Wnt/β-catenin signaling. We hypothesize that USP30-AS1 acts as a molecular sponge for miR-3646, thereby derepressing FZD7, a key Wnt receptor, and amplifying Wnt/β-catenin signaling to promote stemness and chemoresistance in BCSCs. Elucidating this axis may provide a novel therapeutic strategy to concurrently target BCSC stemness and reverse chemoresistance, addressing a critical unmet need in current breast cancer treatment.

## 2. Materials and Methods

### 2.1. Cell Culture and Transfection

Human breast cancer cell lines MCF-7 and MDA-MB-231 were obtained from ATCC. MCF-7 cells were cultured in RPMI-1640 medium supplemented with 10% fetal bovine serum (FBS) and 1% antibiotic-antimycotic. MDA-MB-231 cells were maintained in high-glucose DMEM containing 10% FBS and 1% antibiotic-antimycotic. All cells were grown at 37 °C under 5% CO_2_.

The siRNA and miRNA were synthesized by GenePharma (Shanghai, China). The pcDNA-USP30-AS1 was constructed by Youbio (Changsha, China). Cells at 70–80% confluence were transfected with 1–2 µg plasmid or 50–100 pmol siRNA/miRNA using the jet PRIME transfection reagent according to the manufacturer’s instructions.

### 2.2. Tumor Spheroid Formation Assay

Stem cell spheroids from MCF-7 and MDA-MB-231 cells were generated using a serum-free induction approach combined with gradual serum reduction. Briefly, adherent cells were cultured until 70–80% confluent, trypsinized, and counted. Subsequently, cells were resuspended in serum-free DMEM/F12 medium containing 20 ng/mL bFGF, 20 ng/mL EGF, and 2% B27 supplement at a density of 10^4^ cells/mL, and they were seeded into ultra-low attachment 6-well plates. The plates were maintained at 37 °C for 7 days. Half of the medium was refreshed every 3 days, and spheroids were passaged every 7 days.

### 2.3. Quantitative Real-Time PCR (qRT-PCR)

Total RNA was extracted with Trizol reagent (Invitrogen, Waltham, MA, USA) according to the manufacturer’s instructions. Reverse transcription was performed using 100 ng to 1 µg of total RNA with the TransScript^®^ All-in-One First-Strand cDNA Synthesis SuperMix for qPCR (TransGen, Beijing, China). SYBR-green based real-time PCR was used analyze gene expression (TOYOBO, Osaka, Japan). The relative expression abundance of the target gene was analyzed by the 2^−ΔΔCt^ method using GAPDH or U6 as the endogenous control. The qPCR primer sequences are listed in Table A1.

### 2.4. Transwell Assay

Cell migration and invasion assays were performed using Transwell chambers with 8 μm pore size filters. After serum starvation for 12–24 h, cells were digested and resuspended in serum-free medium. Then, 3 × 10^4^ cells in 200 μL serum-free medium were seeded into the upper chamber, and 600 μL medium containing 15% FBS was added to the lower chamber as a chemoattractant. Cells were incubated for 24 h (migration) or 48 h (invasion) under standard culture conditions. After incubation, non-migrated/invaded cells on the upper surface were carefully removed with a cotton swab. Cells on the lower membrane were fixed with methanol for 30 min, stained with 0.2% crystal violet, and washed with PBS. The number of migrated/invaded cells was counted in five random fields per membrane under a microscope, and experiments were performed in triplicate.

### 2.5. Western Blot

The samples were lysed and protein concentration was determined using a BCA assay kit (Beyotime, # P0006C, Haimen, China). Equal amounts of protein were separated by 10% SDS-PAGE and transferred onto nitrocellulose membranes. After blocking with 5% non-fat milk in TBST for 1 h at room temperature, the membranes were incubated overnight at 4 °C with the following primary antibodies: anti-TCF4 (13838-1-AP, Proteintech, Rosemont, IL, USA), anti-FZD7 (16974-1-AP, Proteintech), anti-AXIN2 (20540-1-AP, Proteintech), anti-CD44 (15675-1-AP, Proteintech), anti-P-gP (22336-1-AP, Proteintech), anti-c-MYC (10828-1-AP, Proteintech), anti-β-catenin (sc-7963, Santa Cruz Biotechnology, Dallas, TX, USA), anti-Cyclin D1 (#2978, Cell Signaling Technology, Danvers, MA, USA), and anti-GAPDH (#10442-1-AP, Proteintech). Subsequently, the membranes were incubated with HRP-conjugated secondary antibodies for 1 h at room temperature. Protein bands were visualized using an ECL detection reagent.

### 2.6. CCK-8 Proliferation Assay

Cells were seeded into 96-well plates at a density of 3 × 10^3^ cells per well in 100 µL of complete medium and allowed to adhere under standard culture conditions. After cell attachment, the medium was replaced with fresh medium containing various concentrations of the test compound, prepared with 0.1% DMSO. Following 24 h or 48 h of incubation at 37 °C, the treatment medium was carefully removed. Each well then received 100 µL of fresh complete medium and 10 µL of CCK-8 reagent, followed by incubation for 1 h at 37 °C. Absorbance was measured at 450 nm using a microplate reader. The half-maximal inhibitory concentration (IC_50_) was calculated based on the recorded data.

### 2.7. Luciferase Reporter Assay

Cells were seeded in 6-well plates and transfected with the Myc-TA-luc reporter plasmid containing TCF/LEF binding sites, along with the pRL-TK plasmid expressing Renilla luciferase for normalization. Where applicable, co-transfection was performed with plasmids for USP30-AS1 knockdown or overexpression. After 48 h, the medium was aspirated and cells were washed once with PBS. Subsequently, 250 µL of 1× Passive Lysis Buffer (PLB, Promega, Madison, WI, USA) was added to each well, and plates were gently rocked for 15 min at room temperature. The lysates were collected and 20 µL of supernatant from each sample was transferred in duplicate or triplicate to a black 96-well plate. Firefly luciferase activity was measured immediately after adding 100 µL of Luciferase Assay Reagent II (Promega, USA) using a microplate reader. Then, 100 µL of Stop & Glo^®^ Reagent (Promega, USA) was added to each well to quench Firefly luciferase activity and simultaneously initiate Renilla luciferase activity. Luminescence signals were recorded sequentially. The relative luciferase activity was expressed as the ratio of Firefly to Renilla luminescence for each sample.

### 2.8. Detection of ALDH Activity Using ALDEFLUOR Assay and Flow Cytometry

ALDH enzymatic activity was detected using the ALDEFLUOR™ Kit (Stemcell Technologies, #01700, Vancouver, BC, Canada) according to the manufacturer’s instructions. Briefly, single-cell suspensions were prepared from mammospheres and resuspended in ALDEFLUOR™ Assay Buffer at a concentration of 1 × 10^6^ cells/mL. The ALDEFLUOR™ substrate was activated prior to use. For each assay, 1 mL of cell suspension was aliquoted into a test tube, and 5 µL of activated ALDEFLUOR™ reagent was added. Immediately, half of this mixture was transferred to a control tube containing 5 µL of diethylaminobenzaldehyde (DEAB) reagent, a specific ALDH inhibitor. Both tubes were incubated at 37 °C for 30–60 min in the dark. After incubation, the cells were centrifuged at 250× *g* for 5 min, the supernatant was discarded, and the cell pellet was resuspended in 0.5 mL of ice-cold ALDEFLUOR™ Assay Buffer. The samples were filtered through a 400-mesh nylon sieve to obtain a single-cell suspension and kept on ice until analysis. Flow cytometry was performed using a CytoFLEX flow cytometer (Beckman Coulter, Brea, CA, USA), and ALDH-positive cells were identified based on fluorescence intensity in the FITC channel, with the DEAB-treated sample serving as the negative control.

### 2.9. Statistical Analysis

All data presented are means ± SD from three independent experiments. Statistical significance was assessed via Student’s *t*-test (two groups), Two-way ANOVA (multiple groups), with a threshold of *p* < 0.05 considered statistically significant. Significance levels are denoted as follows: * *p* < 0.05, ** *p* < 0.01, *** *p* < 0.001, and **** *p* < 0.0001.

## 3. Results

### 3.1. Elevated Expression of USP30-AS1 in Breast Cancer Stem Cells

**Figure 1:**
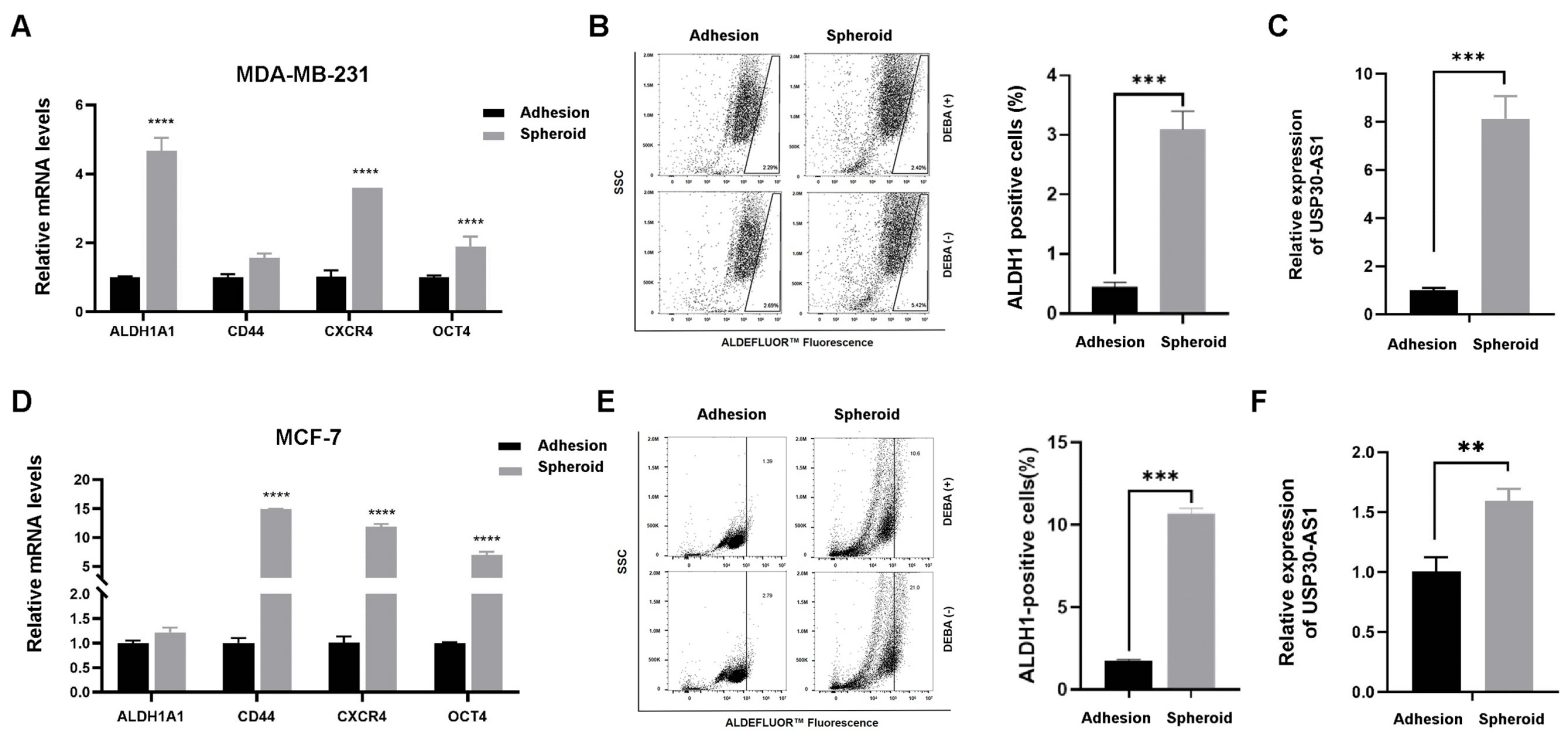
**USP30-AS1 is upregulated in breast cancer stem cells. **(**A**) qPCR analysis of stemness marker expression in MDA-MB-231 adherent cells versus mammospheres derived stem-like cells. (**B**) Flow cytometry analysis of the ALDH1-positive population in wild-type MDA-MB-231 cells versus spheroids. (**C**) qPCR detection of USP30-AS1 expression in MDA-MB-231 spheroids derived stem-like cells. (**D**) The mRNA levels of stemness markers were determined by qPCR in MCF-7 adherent cells versus spheroids-derived stem-like cells. (**E**) Flow cytometry analysis of the ALDH1-positive population in MCF-7 adherent cells versus spheroids. (**F**) USP30-AS1 expression in MCF-7 spheroids-derived stem-like cells was determined by qPCR analysis. The dots on the right side of the line in B and E indicate ALDH-positive cells. ** *p* < 0.01, *** *p* < 0.001, and **** *p* < 0.0001 versus Adhesion.

To investigate the potential role of USP30-AS1 in breast cancer stemness, we generated mammospheres from MDA-MB-231 and MCF-7 cell lines using serum-free suspension culture, a well-established model for enriching breast cancer stem cells (BCSCs). After one week of culture, the spheroids were collected and analyzed. qPCR results demonstrated significant upregulation of key stemness-related markers, including CD44, CXCR4, ALDH1A1, and OCT4, in spheroids from both cell lines compared to their adherent counterparts (Figure 1A,D). Consistent with this, the ALDEFLUOR™ assay revealed a markedly increased proportion of ALDH-positive cells in spheroids (Figure 1B,E), further confirming the successful enrichment of BCSCs. We subsequently evaluated the expression level of USP30-AS1 in these spheroids cultures. Notably, USP30-AS1 expression was substantially elevated approximately 8-fold in MDA-MB-231 and 1.5-fold in MCF-7 spheroids, relative to adherent cells (Figure 1C,F). These results indicate that USP30-AS1 is highly expressed in BCSC-enriched populations and suggest a potential involvement in regulating stemness.

### 3.2. USP30-AS1 Modulates Breast Cancer Stem Cell Properties

**Figure 2:**
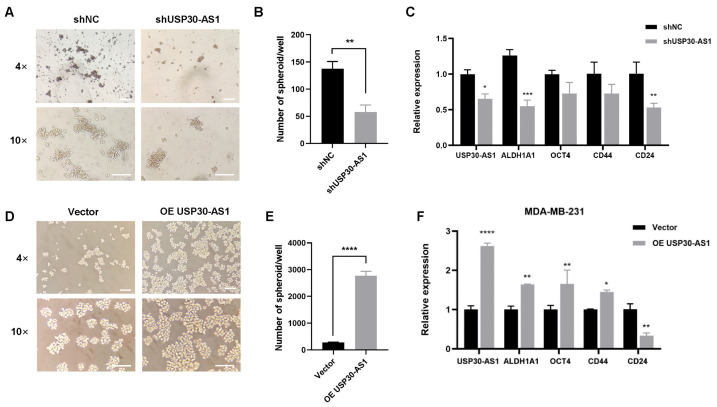
**USP30-AS1 modulates breast cancer stem cells properties.** (**A**) MDA-MB-231 cells were transduced with lentivirus for USP30-AS1 shRNA and subsequently induced to form mammospheres. Representative light microscopy images show the size and number of spheroids formed by USP30-AS1-knockdown cells. Scale bar: 200 μm. (**B**) The number of spheroids per well was quantified. (**C**) qPCR analysis of stemness marker expression following stable knockdown of USP30-AS1. (**D**) MDA-MB-231 cells were transduced with lentivirus for stable overexpression of USP30-AS1 and then induced to form mammospheroids. Light microscopy was used to observe mammospheroid formation. Scale bar: 200 μm. (**E**) The number of spheroids per well was quantified. (**F**) qPCR detection of stemness marker expression in MDA-MB-231 cells stably overexpressing USP30-AS1. * *p* < 0.05, ** *p* < 0.01, *** *p* < 0.001, and **** *p* < 0.0001 versus shNC or Vector.

To determine whether USP30-AS1 upregulation functionally contributes to breast cancer stem cell traits, we genetically modulated its expression in MDA-MB-231 cells using lentivirus-mediated stable knockdown and overexpression. Mammospheroid formation assays were then performed to assess self-renewal capacity. Stable knockdown of USP30-AS1 significantly reduced both the size and number of spheroids compared to control cells (Figure 2A,B). Consistent with this inhibitory effect, qPCR analysis showed that silencing USP30-AS1 markedly downregulated the expression of key stemness-related genes (Figure 2C). Conversely, stable overexpression of USP30-AS1 promoted spheroid formation, resulting in increased spheroid size and number (Figure 2D,E). Furthermore, USP30-AS1 overexpression elevated the mRNA levels of stemness markers and the multidrug resistance gene MDR1 (Figure 2F). These results demonstrate that USP30-AS1 positively regulates BCSC properties, including self-renewal and expression of stemness and resistance markers.

### 3.3. USP30-AS1 Confers Chemoresistance in Breast Cancer Stem Cells

Given the critical role of the multidrug resistance gene *MDR1* (encoding P-glycoprotein, P-gp) in mediating chemoresistance, particularly in BCSCs, we next investigate whether USP30-AS1 influences drug sensitivity. Using doxorubicin, a standard chemotherapeutic agent in breast cancer treatment, we performed CCK-8 assays to evaluate cell viability under different genetic manipulations of USP30-AS1.

**Figure 3:**
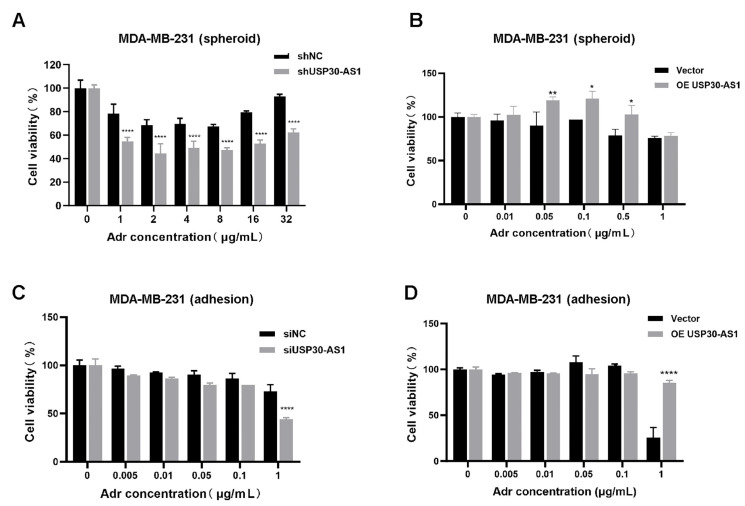
**USP30-AS1 reduces the chemosensitivity in BCSCs.** (**A**) Viability of MDA-MB-231-derived mammospheroid cells stably knockdown or (**B**) overexpression of USP30-AS1 after treatment with various concentrations of doxorubicin for 24 h, as determined by CCK-8 assay. (**C**) Effect of siRNA-mediated knockdown or (**D**) overexpression of USP30-AS1 on the sensitivity of MDA-MB-231 adherent cells to doxorubicin, as determined by CCK-8 assay after 24 h treatment. * *p* < 0.05, ** *p* < 0.01, and **** *p* < 0.0001 versus shNC, siNC or Vector.

In BCSC-enriched models, stable knockdown of USP30-AS1 significantly enhanced cellular sensitivity to doxorubicin (Figure 3A). Conversely, stable overexpression of USP30-AS1 reduced drug sensitivity (Figure 3B). To determine whether this effect is specific to the stem cell subpopulation or generalizable to bulk tumor cells, we extended our analysis to adherent cells. Consistent with the BCSC results, siRNA-mediated knockdown of USP30-AS1 increased doxorubicin sensitivity in adherent cultures, whereas overexpression of USP30-AS1 decreased it (Figure 3C,D). These findings indicate that USP30-AS1 contributes broadly to chemoresistance in both stem-like and bulk populations of breast cancer cells, and underscores its role as a potential modulator of treatment response.

### 3.4. USP30-AS1 Enhances Migratory, Invasive Abilities and WNT Transcriptional Activity

**Figure 4:**
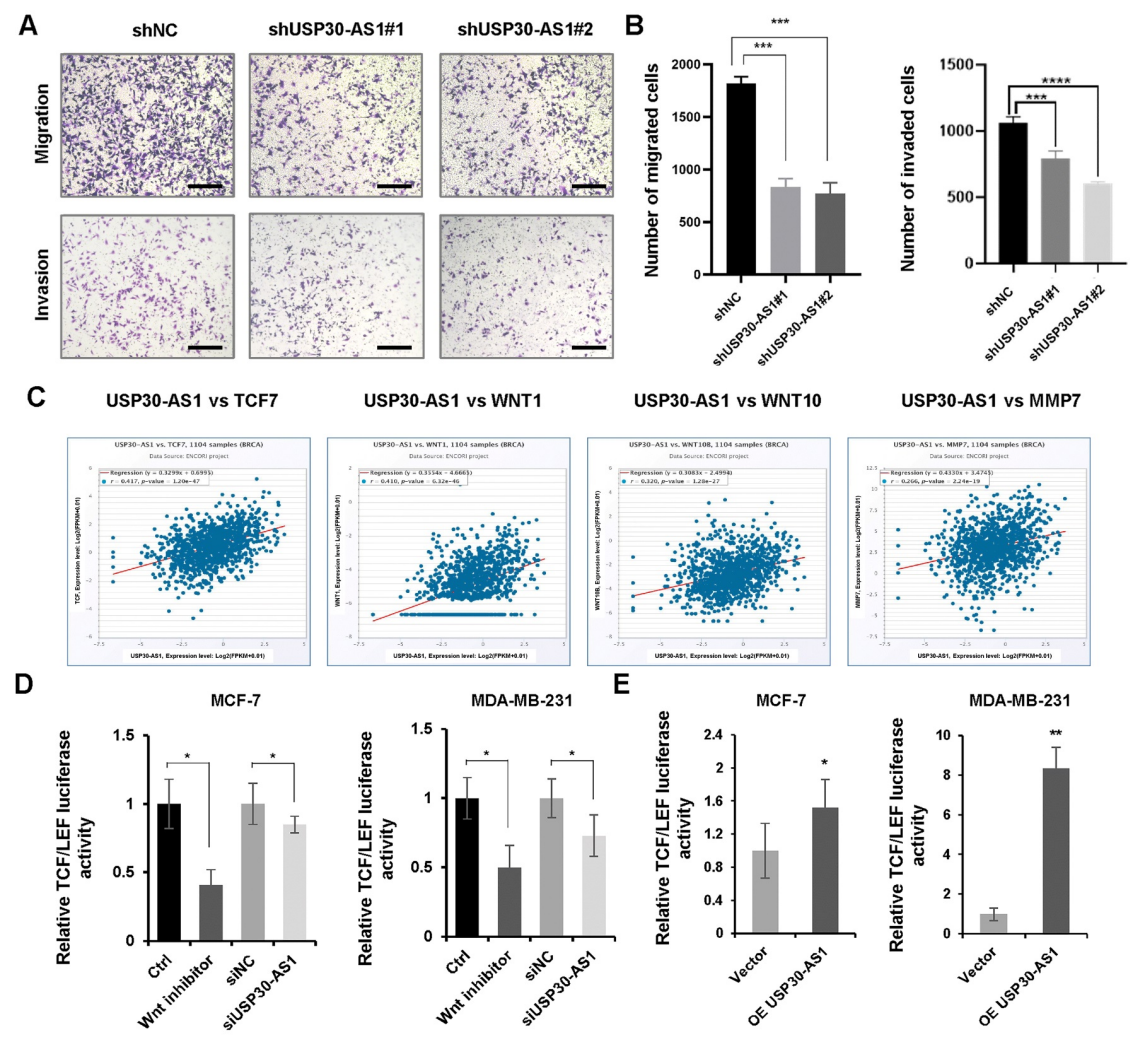
**USP30-AS1 promotes breast cancer cell invasion and migration by enhancing WNT transcriptional activity.** (**A**) Transwell migration and invasion assays evaluating the effect of stable USP30-AS1 knockdown with shRNA in breast cancer cells. Scale bar: 100 μm. (**B**) Quantitative analysis of the number of migrated and invaded cells. (**C**) Correlation analysis between USP30-AS1 expression and Wnt pathway-related genes (TCF7, WNT1, WNT10, MMP7) in breast cancer using cBioPortal. (**D**) Dual-luciferase reporter assay assessing TCF/LEF transcriptional activity in MDA-MB-231 and MCF-7 cells treated with control, Wnt inhibitor, non-targeting control siRNA, or USP30-AS1-targeting siRNA. (**E**) Luciferase reporter assay evaluating the effect of vector or USP30-AS1 overexpression on Wnt pathway transcriptional activity in MDA-MB-231 and MCF-7 cells. * *p* < 0.05, ** *p* < 0.01, *** *p* < 0.001, and **** *p* < 0.0001 versus shNC, Ctrl, siNC or Vector.

It is well established that cancer stem cells are pivotal drivers of tumor metastasis and therapeutic resistance [37,38]. To evaluate the role of USP30-AS1 in metastatic potential, we performed Transwell migration and invasion assays. The results demonstrated that knockdown of USP30-AS1 significantly suppressed both migratory and invasive capacities of breast cancer cells (Figure 4A,B). Bioinformatic analysis using cBioPortal v6.0.0, revealed a positive correlation between USP30-AS1 expression and several Wnt signaling components, including TCF7, WNT1, WNT10, and MMP7, in clinical breast cancer (Figure 4C). Given the critical role of Wnt signaling in CSC self-renewal and metastasis, we assessed its transcriptional activity using a TCF/LEF luciferase reporter system. Luciferase assays showed that USP30-AS1 knockdown markedly reduced Wnt reporter activity, while its overexpression enhanced transcriptional activation (Figure 4D,E).

### 3.5. USP30-AS1 Modulates Expression of Key Wnt Pathways Components

**Figure 5:**
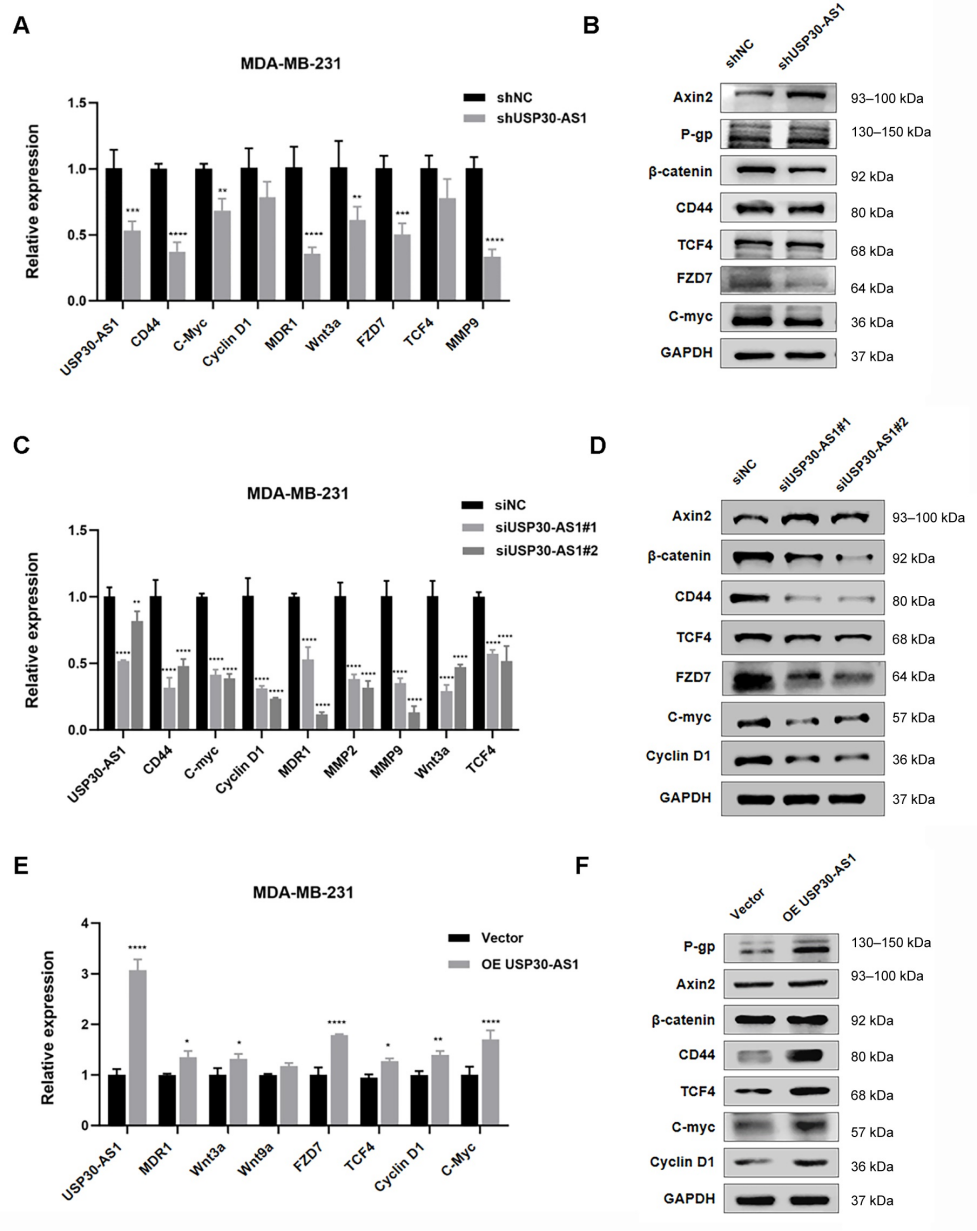
**USP30-AS1 modulates the expression of Wnt-related genes.** (**A**) qPCR analysis of mRNA expression levels of key Wnt pathway-related factors in MDA-MB-231 cells stably transfected with shRNA targeting USP30-AS1. Data are presented as mean ± SD. (**B**) Western blot analysis of Wnt-related proteins in stable USP30-AS1-knockdown MDA-MB-231 cells. GAPDH was used as a loading control. (**C**) qPCR detection of Wnt pathway gene expression following transient transfection with siRNA against USP30-AS1. Data are presented as mean ± SD. (**D**) Western blot validation of Wnt signaling components after transient siRNA-mediated knockdown of USP30-AS1. (**E**) qPCR evaluation of Wnt target genes in MDA-MB-231 cells overexpressing USP30-AS1. Data are presented as mean ± SD. (**F**) Western blot analysis confirming upregulation of Wnt pathway factors upon USP30-AS1 overexpression.* *p* < 0.05, ** *p* < 0.01, *** *p* < 0.001, and **** *p* < 0.0001 versus shNC, siNC or Vector.

To further investigate the mechanistic basis of Wnt activation by USP30-AS1, we analyzed the expression of core Wnt-related factors following genetic modulation of USP30-AS1. Stable knockdown using shRNA significantly downregulated the mRNA and protein levels of multiple Wnt pathway genes, including TCF4, MMP9, WNT9a, c-Myc, cyclin D1, and MDR1, as confirmed by qPCR and Western blotting (Figure 5A,B). Similarly, transient transfection with two independent siRNAs targeting USP30-AS1 consistently reduced the expression of these Wnt signaling components compared to negative control (Figure 5C,D). Conversely, stable overexpression of USP30-AS1 of USP30-AS1 increased both transcript and protein levels of these genes (Figure 5E,F). These results indicate that USP30-AS1 functions as a positive regulator of Wnt/β-catenin signaling, contributing to the maintenance of an invasive and chemoresistant phenotype in breast cancer.

### 3.6. USP30-AS1 Regulates WNT Signaling via Sponging miR-3646

**Figure 6:**
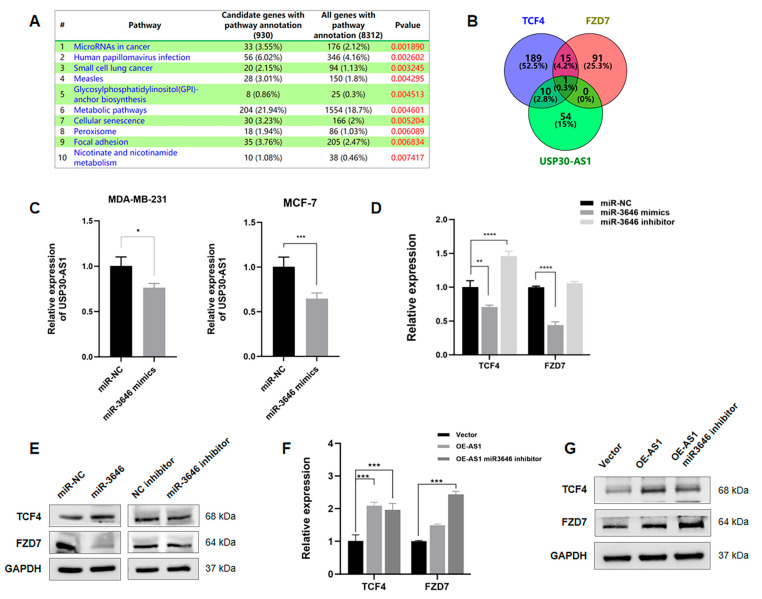
**USP30-AS1 regulates WNT signaling via sponging miR-3646.** (**A**) Gene Set Enrichment Analysis (GSEA) of RNA-seq data from USP30-AS1-knockdown versus control cells. The “MicroRNAs in Cancer” pathway was identified as the most significantly enriched gene set. (**B**) Venn diagram analysis of predicted miRNAs targeting USP30-AS1, TCF4, and FZD7 (based on miRDB predictions with score > 60). miR-3646 was identified as the common miRNA targeting all four genes. (**C**) qPCR analysis of USP30-AS1 expression in MDA-MB-231 and MCF-7 cells after transfection with miR-3646 mimics. (**D**) qPCR detection of TCF4 and FZD7 mRNA expression in MDA-MB-231 cells transfected with negative control (NC), miR-3646 mimics, or miR-3646 inhibitor. (**E**) Western blot analysis of TCF4 and FZD7 protein levels in cells transfected with miR-3646 mimics or inhibitor. (**F**) qPCR and (**G**) Western blotting analyses of TCF4 and FZD7 expression in MDA-MB-231 cells transfected with vector, USP30-AS1 overexpression plasmid, or USP30-AS1 plasmid together with miR-3646 inhibitor.* *p* < 0.05, ** *p* < 0.01, *** *p* < 0.001, and **** *p* < 0.0001 versus miR-NC or Vector.

To elucidate the molecular mechanism by which USP30-AS1 regulates breast cancer stem cells, RNA sequencing was performed on control and USP30-AS1 silenced breast cancer cells. Gene Set Enrichment Analysis (GSEA) of the differentially expressed genes identified the top ten enriched pathways, among which “MicroRNAs in Cancer” was the most significantly enriched. This pathway included multiple Wnt-related genes such as WNT3, WNT9, Axin2, FZD7, and TCF4 (Figure 6A). Given the previously established cytoplasmic localization of USP30-AS1 and the known function of cytosolic lncRNAs as miRNA sponges, we hypothesized that USP30-AS1 might post-transcriptionally regulate Wnt signaling by sequestering miRNAs. To test this, potential miRNA targets of USP30-AS1, TCF4 and FZD7 were predicted using miRDB. Venn diagram analysis revealed miR-3646 as the common miRNA targeting all three genes (Figure 6B).

Functional validation was conducted using miR-3646 mimics and inhibitors. qPCR analysis demonstrated that miR-3646 overexpression downregulated the expression of USP30-AS1, TCF4, and FZD7, whereas inhibition of miR-3646 elevated their expression (Figure 6C,D). Western blot analysis further confirmed that miR-3646 mimics reduced FZD7 protein levels, which was restored by the miR-3646 inhibitor. In contrast, TCF4 protein expression was not significantly affected by miR-3646 modulation (Figure 6E). Rescue experiments showed that overexpression of USP30-AS1 increased both TCF4 and FZD7 expression. Moreover, co-transfection with the miR-3646 inhibitor further enhanced FZD7 levels (Figure 6F,G). The additive effect observed when USP30-AS1 is overexpressed together with the miR-3646 inhibitor provide direct evidence that USP30-AS1 functions by sequestering miR-3646, thereby preventing it from repressing its target FZD7. Collectively, these results suggest that USP30-AS1 promotes Wnt signaling through USP30-AS1/miR-3646/FZD7 ceRNA axis.

## 4. Discussion

This study demonstrates that the long non-coding RNA USP30-AS1 plays a critical role in promoting stemness, chemoresistance, migration and invasion in BSCSs through activation of the Wnt/β-catenin signaling pathway. We found that USP30-AS1 is upregulated in BCSC-enriched mammospheroids derived from MDA-MB-231 and MCF-7 cells, consistent with elevated progression of stemness markers (CD44, CXCR4, ALDH1A1, OCT4) and an increased proportion of ALDH-positive cells. Functional assays established that USP30-AS1 knockdown suppresses spheroid formation, stemness marker expression, migration, invasion, and doxorubicin resistance, while its overexpression enhances these effects. Importantly, we identified a novel molecular mechanism whereby cytoplasmic USP30-AS1 functions as a competing endogenous RNA by sequestering miR-3646, leading to the derepression of FZD7 and subsequent activation of Wnt/β-catenin signaling.

Our previous study reported the upregulation and oncogenic role of USP30-AS1 in breast cancer via the HnRNPF/p21 and EZH2/c-Myc/p21 axes [31]. Here, we specifically explore its function in BCSCs, a subpopulation with critical roles in therapy resistance and recurrence. Given the established role of the Wnt pathway in regulating BCSC self-renewal and chemoresistance [34,39], we sought to determine whether USP30-AS1 activates this pathway. We provide mechanistic evidence that USP30-AS1 acts as an upstream activator of Wnt signaling pathway through post-transcriptional regulation. Specifically, we identified miR-3646 as a direct of USP30-AS1 and demonstrated that USP30-AS1 sequesters miR-3646 to suppress FZD7 expression. miR-3646 has been reported to act as a potential oncogene in lung adenocarcinoma and breast cancer [40,41] and to contribute to docetaxel resistance in breast cancer cells by suppressing the GSK-3β/β-catenin signaling pathway [42]. Our results position the USP30-AS1/miR-3646/FZD7 axis as a central regulatory node in BCSC maintenance.

This study also adds significant insight into the growing understanding of lncRNA-mediated miRNA sponging in BCSC regulation. The importance of lncRNA-miRNA functional axes in breast cancer pathogenesis has been increasingly recognized, revealing complex regulatory networks that influence tumor progression and stemness [43,44]. Several lncRNAs have been demonstrated to employ similar ceRNA mechanisms in modulating BCSC properties. For instance, lncRNA CASC15 sustains breast cancer stemness through the miR-654-5p/MEF2D axis [45], while HAGLROS promotes tumor evolution via the miR-135b-3p/COL10A1 pathway and exosome-mediated macrophage polarization [46]. In triple-negative breast cancer, LINC00273 enhances metastasis and stemness through miRNA sponging [47], and H19 modulates Wnt signaling by sponging miR-141 [48]. In line with these findings, our rescue experiments identify USP30-AS1 as a novel ceRNA that drives BCSC properties through its specific sequestration of miR-3646 and subsequent regulation of FZD7, highlighting the complexity of non-coding RNA networks fine-tuning BCSC properties.

Building upon this work, several promising directions need further investigation. First, while out study clarifies the cytoplasmic ceRNA mechanism of USP30-AS1 in BCSCs, its nuclear roles such as potential interactions with epigenetic regulators like EZH2, as identified in our previous work, remained to be explored in the context of stemness [31]. Future studies should examine whether USP30-AS1 exerts compartment-specific or coordinated functions across nuclear and cytoplasmic domains to regulate BCSC properties. Second, the upstream signals that induce USP30-AS1 overexpression in BCSCs, such as hypoxia, inflammatory cytokines, or metabolic stress, are still unknown. Identifying these triggers will provide deep insight into the regulation of this oncogenic lncRNA within the tumor microenvironment. Finally, while our study established the role of USP30-AS1 in doxorubic resistance, its impact on other therapeutic agents, including taxanes or PARP inhibitors, represents an important area for further investigation. The use of more complex models, such as patient-derived organoids or xenografts, will help validate the clinical relevance of the USP30-AS1/miR-3646/FZD7 axis and assess its targetability in vivo.

Together, our work establishes that USP30-AS1 drives BCSC properties via the miR-3646/FZD7/Wnt axis, representing a promising therapeutic target for disrupting BCSC maintenance and reversing chemoresistance. Targeting this axis using antisense oligonucleotides (ASOs) against USP30-AS1 or miR-3646 mimetics may effectively suppress breast cancer stemness and resensitize tumors to conventional chemotherapy.

## 5. Conclusions

In conclusion, we have identified a novel USP30-AS1/miR-3646/FZD7 regulatory circuit that promotes stemness, chemoresistance, migration and invasion in BCSCs via activation of Wnt/β-catenin signaling. These results not only deepen our understanding of lncRNA-mediated regulatory mechanisms in BCSCs but also provide a preclinical rationale for targeting this axis as a therapeutic strategy in breast cancer.

## Data Availability

The original contributions presented in this study are included in the article. Further inquiries can be directed to the corresponding authors. Human breast cancer cell lines MCF-7 and MDA-MB-231 were obtained from ATCC.

## Appendix A

**Table A1:** List of primers used for qRT-PCR analyses.

Gene	Sense (5′-3′)	Antisense (5′-3′)
USP30-AS1	CCAGAGTGGAAATAGGTCGCA	GGCACCCAAGTAAACAATAAGT
CD44	CTGCCGCTTTGCAGGTGTA	CATTGTGGGCAAGGTGCTATT
MDR1	GCTCCTGACTATGCCAAAGCC	CTTCACCTCCAGGCTCAGTCCC
OCT4	GGGAGATTGATAACTGGTGTGTT	GTGTATATCCCAGGGTGATCCTC
CXCR4	ACTACACCGAGGAAATGGGCT	CCCACAATGCCAGTTAAGAAGA
ALDH1A1	GCACGCCAGACTTACCTGTC	CCTCCTCAGTTGCAGGATTAAAG
C-myc	CTTCTCTCCGTCCTCGGATTCT	GAAGGTGATCCAGACTCTGACCTT
Cyclin D1	GGCGGATTGGAAATGAACTT	TCCTCTCCAAAATGCCAGAG
TCF4	GCCTCTTATCACGTACAGCAAT	GCCAGGCGATAGTGGGTAAT
Axin2	ATTCGGCCACTGTTCAGACG	GACAACCAACTCACTGGCCTG
FZD7	TTCTCGGACGATGGCTACC	GAACCAAGTGAGAGACAGAATGACC
β-catenin	TGGATTGATTCGAAATCTTGC	GAACAAGCAACTGAACTAGT
Wnt3a	GCACCACCGTCCACGACAGC	CCTCGCTACAGCCACCCCAC
Wnt9a	TGGAGGCCGTGAGCATGAGT	CTTAAGGTTGTCTCCGCAGC
GAPDH	UGAGGCAGUAGAUUGAAU	UCAAUCUACUGCCUCAUU
